# Investigation of quadratic electro-optic effects and electro-absorption process in GaN/AlGaN spherical quantum dot

**DOI:** 10.1186/1556-276X-9-131

**Published:** 2014-03-19

**Authors:** Mohammad Kouhi, Ali Vahedi, Abolfazl Akbarzadeh, Younes Hanifehpour, Sang Woo Joo

**Affiliations:** 1 Department of Physics, College of Science, Tabriz Branch, Islamic Azad University, Tabriz 5157944533, Iran; 2 Department of Medical Nanotechnology, Faculty of Advanced Medical Science, Tabriz University of Medical Sciences, Tabriz 5154853431, Iran; 3 School of Mechanical Engineering, Yeungnam University, Gyeongsan 712-749, South Korea

**Keywords:** Quadratic electro-optic effects, Third-order susceptibility, Spherical quantum dot, Relaxation time

## Abstract

Quadratic electro-optic effects (QEOEs) and electro-absorption (EA) process in a GaN/AlGaN spherical quantum dot are theoretically investigated. It is found that the magnitude and resonant position of third-order nonlinear optical susceptibility depend on the nanostructure size and aluminum mole fraction. With increase of the well width and barrier potential, quadratic electro-optic effect and electro-absorption process nonlinear susceptibilities are decreased and blueshifted. The results show that the DC Kerr effect in this case is much larger than that in the bulk case. Finally, it is observed that QEOEs and EA susceptibilities decrease and broaden with the decrease of relaxation time.

## Background

Semiconductor quantum dots with their excellent optoelectronic properties are now mostly used for various technologies such as biological science [1-4], quantum dot lasers [5,6], light-emitting diodes (LEDs) [7], solar cells [8], infrared and THZ-IR photodetectors [9-14], photovoltaic devices [15], and quantum computing [16,17]. GaN and AlN are members of III-V nitride family. These wide bandgap semiconductors are mostly appropriate for optoelectronic instrument fabrication.

Third-order nonlinear optical processes in ZnS/CdSe core-shell quantum dots are investigated in [18-20]. It is shown that the symmetry of the confinement potential breaks due to large applied external electric fields and leads to an important blueshift of the peak positions in the nonlinear optical spectrum. The effect of quantum dot size is also studied, and it is verified that large nonlinear third-order susceptibilities can be achieved by increasing the thickness of the nanocrystal shell.

The authors of [21,22] studied the quadratic electro-optic effects (QEOEs) and electro-absorption (EA) process in InGaN/GaN cylinder quantum dots and CdSe-ZnS-CdSe nanoshell structures. They have found that the position of nonlinear susceptibility peak and its amplitude may be tuned by changing the nanostructure configuration. The obtained susceptibilities in these works are around $10^{-17}\frac{m^{2}}{v^{2}}$ and 10^-15^ esu, respectively.

In reference [23], self-focusing effects in wurtzite InGaN/GaN quantum dots are studied. The results of this paper show that the quantum dot size has an immense effect on the nonlinear optical properties of wurtzite InGaN/GaN quantum dots. Also, with decrease of the quantum dot size, the self-focusing effect increases.

In a recent paper [24], we have shown that with the control of GaN/AlGaN spherical quantum dot parameters, different behaviors are obtained. For example, with the increase of well width, third-order susceptibility decreases. The aim of this study is to investigate our proposed GaN/AlGaN quantum dot nanostructure from quadratic electro-optic effect and electro-absorption process points of view. In this paper, we study third-order nonlinear susceptibility of GaN/AlGaN semiconductor quantum dot based on the effective mass approximation. The numerical results have shown that in the proposed structure, the third-order nonlinear susceptibilities near 2 to 5 orders of magnitudes are increased.

The organization of this paper is as follows. In the 'Methods’ section, the theoretical model and background are described. The 'Results and discussion’ section is devoted to the numerical results and discussion. Summarization of numerical results is given in the last section.

## Methods

**Figure 1:**
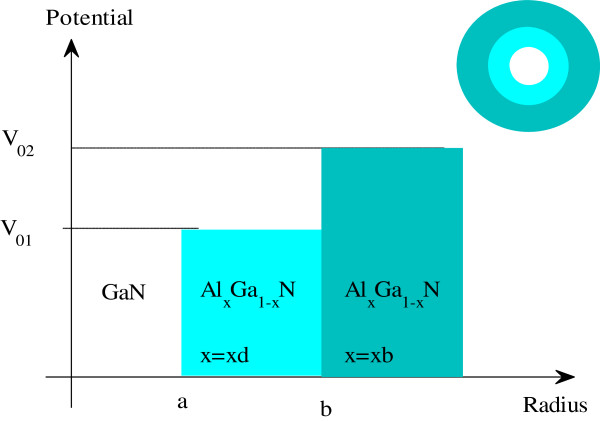
Structure of the spherical quantum dot and related potential distribution.

In this section, theoretical model and mathematical background of the third-order nonlinear properties of a new GaN/AlGaN quantum dot nanostructure are presented. The geometry of a spherical centered defect quantum dot and potential distribution of this nanostructure are shown in Figure 1. We consider three regions consisting of a spherical well (with radius *a*), an inner defect shell (with thickness *b* - *a*), and an outer barrier (with radius *b*). The proposed spherical centered defect quantum dot can be performed by adjusting the aluminum mole fraction.

In this paper, the potential in the core region is supposed to be zero, and the potential difference between two materials is constant [25]. There are various methods for investigating electronic structures of quantum dot systems [26-28]. The effective mass approximation is employed in this study. The time-independent Schrödinger equation of the electron in spherical coordinate can be written as [29].

(1) $$\begin{array}{l} -\frac{\hbar^{2}}{2m_{i}^{*}}\left[\frac{1}{r^{2}}\;\frac{\partial }{\partial r}\left(r^{2}\frac{\partial \psi }{\partial r}\right)+\frac{1}{r^{2}\sin\theta }\frac{\partial }{\partial \theta }\left(\sin\theta \frac{\partial \psi }{\partial \theta }\right)\right)\;\\ \left(+\frac{1}{r^{2}\sin^{2}\theta }\frac{\partial^{2}\psi }{\partial \varphi^{2}}\right]+V_{i}\left(r\right)\;\psi =\mathrm{E\psi}, \end{array}$$

where *m*_*i*_^∗^ and *V*_*i*_(*r*) are effective mass and potential distribution in different regions. They are obtained as follows [30]:

(2) $$m_{i}^{*}=\left\{\begin{array}{l} m_{1}^{*}=0.228m_{e}\;0<r<a\;\\ m_{2}^{*}=\left(0.252\mathrm{xd}+0.228\right)m_{e}\;a<r<b\;\\ m_{3}^{*}=\left(0.252\mathrm{xb}+0.228\right)m_{e}\;b<r\; \end{array}\right)$$

and

(3) $$\begin{array}{l} V_{i}\left(r\right)=\left\{\begin{array}{l} 0\;0<r<a\;\\ V_{01}=\Delta E_{c}\left(\mathrm{xd}\right)\;a<r<b\;\\ V_{02}=\Delta E_{c}\left(\mathrm{xb}\right)\;b<r, \end{array}\right) \end{array}$$

where *xd* and *xb* are defect and barrier regions of aluminum molar fraction, respectively. The rest mass of electron is denoted by *m*_e_, and *ΔE*_c_(*x*) = 0.7 × [*E*_g_(*x*) - *E*_g_(0)] is the conduction band offset [30]. The bandgap energy of Al_*x*_Ga_1 - *x*_N is *E*_g_(*x*) = 6.13*x* + (1 - *x*)(3.42 - *x*) (expressed in electron volts) [30,31]. In a spherical coordinate, Schrödinger Equation 1 can be readily solved with the separation of variables. Thus, the wave function can be written as

(4) ψnℓmr,θ,ϕ=RrnℓYℓmθ,ϕ,

where *n* is the principal quantum number, and *ℓ* and *m* are the angular momentum numbers. *Y*_*ℓm*_(*θ*, *ϕ*) is the spherical harmonic function and is the solution of the angular part of the Schrödinger equation. By substituting Equation 4 into Equation 1, the following differential equation is obtained for *R*_*nℓ*_(*r*):

(5) r2d2Rrnℓdr2+2rdRrnℓdr+2mi*ℏ2E-Virr2-ℓℓ+1Rrnℓ=0

In order to calculate *R*_*nℓ*_(*r*), the two *E* < *V*_01_ and *E* > *V*_01_ cases must be considered. With change of variables and some mathematical rearranging, the following spherical Bessel functions in both cases are obtained:

Case 1: *E* < *V*_01_.

(6) $$R\left(r\right)=\left\{\begin{array}{l} {}[C_{1}j_{\ell }\left(k_{1}r\right)+C_{2}n_{\ell }(k_{1}r)]\sqrt{\frac{2}{\pi }}\;0<r<a\;\\ {}[\;C_{3}i_{\ell }\left(k_{2}r\right)]\sqrt{\frac{2}{\pi }}\;a<r<b\;\\ {}[C_{4}K_{\ell }\left(k_{3}r\right)]\sqrt{\frac{\pi }{2}}\;b<r, \end{array}\right)$$

where

$$\left\{\begin{array}{l} k_{1}=\sqrt{\frac{2m_{1}^{*}E}{\hbar^{2}}}\;0<r<a\;\\ k_{2}=\sqrt{\frac{2m_{2}^{*}\left(V_{01}-E\right)}{\hbar^{2}}}\;a<r<b\;\\ k_{3}=\sqrt{\frac{2m_{3}^{*}\left(V_{02}-E\right)}{\hbar^{2}}}\;b<r\; \end{array}\right)$$

Case 2: *E* > *V*_01_.

(7) $$R\left(r\right)=\left\{\begin{array}{l} {}[C_{1}j_{\ell }\left(k_{1}r\right)+C_{2}n_{\ell }(k_{1}r)]\sqrt{\frac{2}{\pi }}\;0<r<a\;\\ {}[\;C_{3}i_{\ell }\left(k_{2}^{\prime }r\right)]\sqrt{\frac{2}{\pi }}\;a<r<b\;\\ {}[C_{4}K_{\ell }\left(k_{3}r\right)]\sqrt{\frac{\pi }{2}}\;b<r, \end{array}\right)$$

where

$$k_{2}^{\prime }=\sqrt{\frac{2m_{2}^{*}\left(E-V_{01}\right)}{\hbar^{2}}}$$

For the whole determination of eigenenergies and constants that appeared in the wave function, *R*_*nℓ*_(*r*) should satisfy the following boundary, convergence, and normalization conditions.

(8) $$\left\{\begin{array}{l} R_{0<r<a}\left(a\right)=R_{a<r<b}(a)\\ \frac{1}{m_{1}^{*}}{\left(\frac{d}{\mathrm{dr}}\left(R_{0<r<a}\left(r\right)\right)\;\right|}_{r=a}=\frac{1}{m_{2}^{*}}{\left(\frac{d}{\mathrm{dr}}\left(R_{a<r<b}\left(r\right)\right)\;\right|}_{r=a} \end{array}\right)$$

(9) $$\left\{\begin{array}{l} R_{a<r<b}\left(b\right)=R_{b<r}(b)\\ \frac{1}{m_{2}^{*}}{\left(\frac{d}{\mathrm{dr}}\left(R_{a<r<b}\left(r\right)\right)\;\right|}_{r=b}=\frac{1}{m_{3}^{*}}{\left(\frac{d}{\mathrm{dr}}\left(R_{b<r}\left(r\right)\right)\;\right|}_{r=b} \end{array}\right)$$

(10) $$\int_{0}^{\infty }\mathrm{dr}\;r^{2}R_{\mathrm{n\ell}}^{2}\left(r\right)=1$$

After determining the eigenvalues and wave functions, the third-order susceptibility for two energy levels, ground and first excited states, the model should be described [32,33]. Thus, the density matrix method [34,35] is used, and the nonlinear third-order susceptibility corresponding to optical mixing between two incident light fields with frequencies *ω*_1_ and *ω*_2_ appears in Equation 11:

(11) $$\begin{array}{l} \chi^{\left(3\right)}\left(-2\omega_{1}+\omega_{2};\omega_{1},\omega_{1},-\omega_{2}\right)=\frac{-2\mathrm{iN}q^{4}{\left|\alpha_{\mathrm{fg}}\right|}^{4}}{\epsilon_{\circ }\hbar^{3}}\\ \times \left[\frac{1}{\left[i\left(\omega_{\circ }-2\omega_{1}+\omega_{2}\right)+\Gamma ][i\left(\omega_{2}-\omega_{1}\right)+\Gamma \right]}\right]\\ \times \left[\frac{1}{i\left(\omega_{\circ }-\omega_{1}\right)+\Gamma }+\frac{1}{i\left(\omega_{2}-\omega_{\circ }\right)+\Gamma }\right], \end{array}$$

where *q* is electron charge, *N* is carrier density, *α*_fg_ = 〈*ψ*_f_|*r*|*ψ*_g_〉 indicates the dipole transition matrix element, *ω*_o_ = (*E*_f_ - *E*_g_)/*ħ* is the resonance frequency between the first excited and ground states (transition frequency), and Γ is the relaxation rate. For the calculation of third-order susceptibility of QEOEs, we take *ω*_1_ = 0, *ω*_2_ = -*ω* in Equation 11. The third-order nonlinear optical susceptibility *χ*^(3)^(-*ω*, 0, 0, *ω*) is a complex function. The nonlinear quadratic electro-optic effect (DC-Kerr effect) and EA frequency dependence susceptibilities are related to the real and imaginary part of *χ*^(3)^(-*ω*, 0, 0, *ω*) [20-22].

(12) χQEOE3ω=Re[χ3(-ω,0,0,ω)]χEA3ω=Im[χ3(-ω,0,0,ω)]

These nonlinear susceptibilities are important characteristics for photoemission or detection applications of quantum dots.

## Results and discussion

In this section, numerical results including the quadratic electro-optic effect and electro-absorption process nonlinear susceptibilities of the proposed spherical quantum dot are explained. In our calculations, some of the material parameters are taken as follows. The number density of carriers is *N* = 1 × 10^24^ m^-3^, electrostatic constant is *ϵ* = (-0.3*x* + 10.4)*ϵ*_o_[30,31], and typical relaxation constants are *ℏΓ* = 0.27556 and 2.7556 meV which correspond to 15- and 1.5-ps relaxation times, respectively.

**Figure 2:**
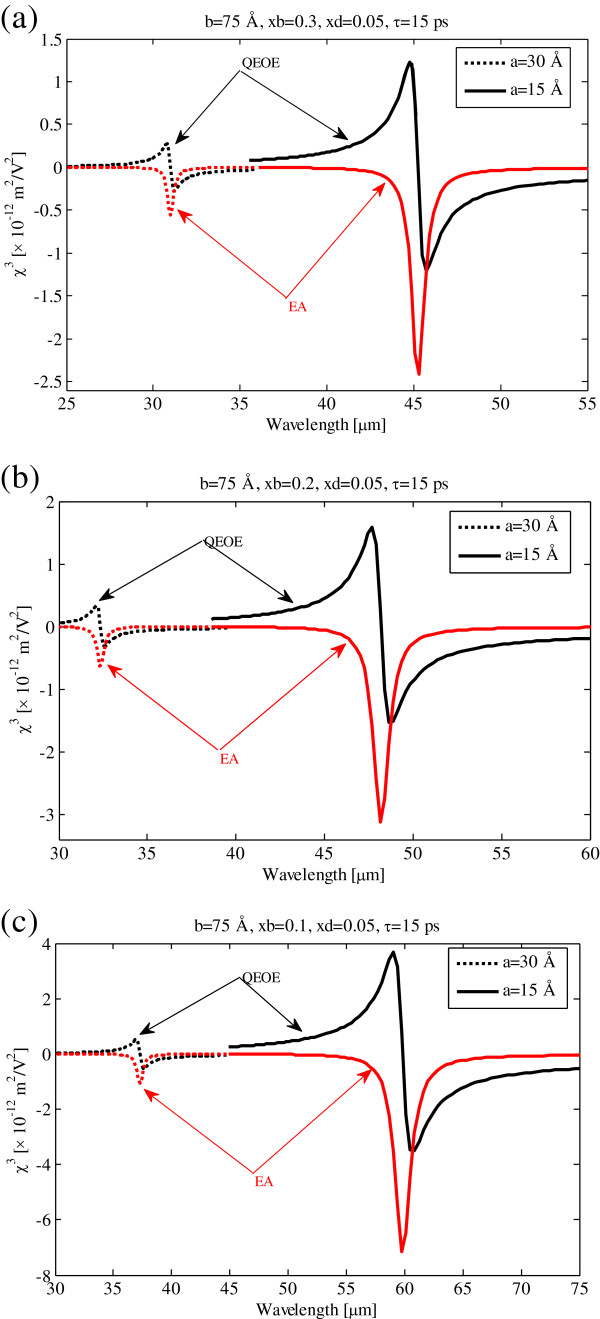
**Quadratic electro-optic effect and electro-absorption process susceptibilities versus pump photon wavelength.** For 15-ps relaxation time, *V*_01_ = 0.062 eV. **(a)***V*_02_ = 0.423 eV. **(b)***V*_02_ = 0.268 eV. **(c)** V_02_ = 0.127 eV.

The quadratic electro-optic effect and electro-absorption process susceptibilities as functions of pump photon wavelength at 15-ps relaxation time are illustrated in Figure 2. In these figure, the solid and dashed lines show 15- and 30-Å well widths, respectively. It is clear that with the increase of the well width, both QEOEs and EA susceptibilities decreased and blueshifted. These behaviors can be related to quantum confinement effect. Because of the increase of well width, the centered defect acts as small perturbation.

**Figure 3:**
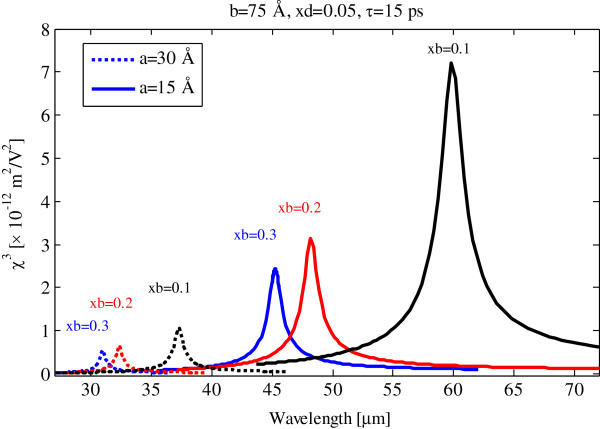
**Third-order susceptibility of GaN/AlGaN quantum dot versus pump photon wavelength.** With different barrier potentials and defect sizes for 15-ps relaxation time.

The third-order susceptibility of GaN/AlGaN quantum dot versus pump photon wavelength with different barrier potentials as parameter is shown in Figure 3. The third-order susceptibility is decreased and blueshifted by the increasing barrier potential. These are related to energy levels and dipole transition matrix element behaviors by dot potential. See Figures four and twelve of [24]. So, the resonance wavelength and magnitude of the third-order susceptibility can be managed by the control of well width and confining quantum dot potential.

**Figure 4:**
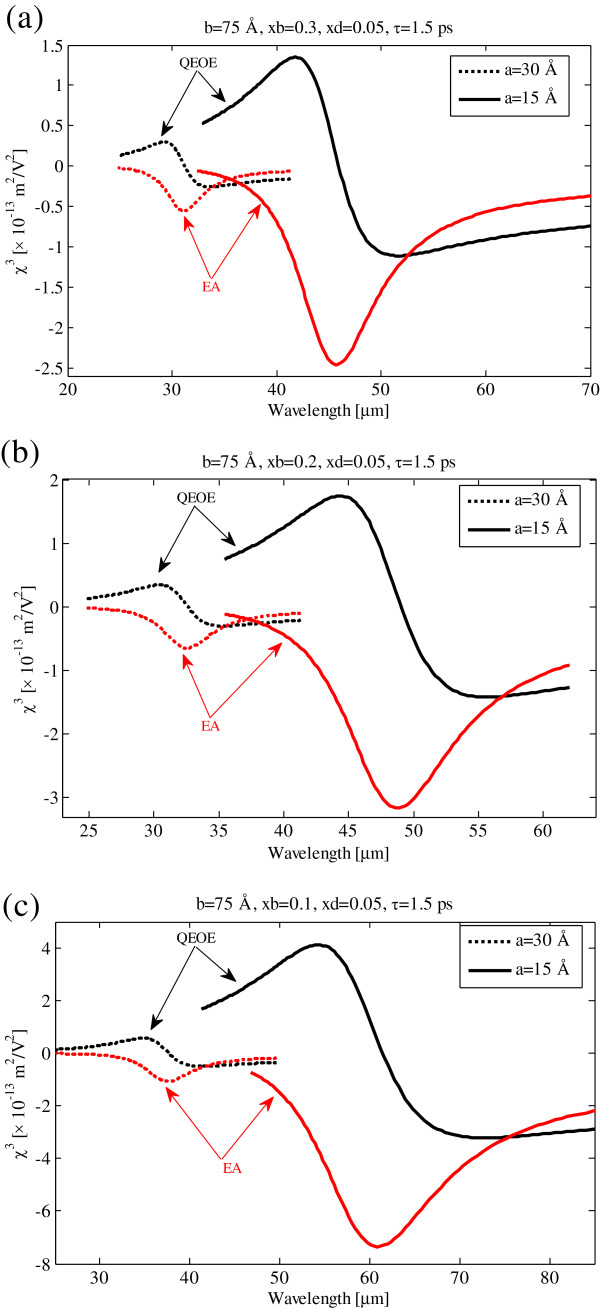
**Quadratic electro-optic effect and electro-absorption process susceptibilities versus pump photon wavelength.** For 1.5-ps relaxation time, *V*_01_ = 0.062 eV. **(a)***V*_02_ = 0.423 eV. **(b)***V*_02_ = 0.268 eV. **(c)***V*_02_ = 0.127 eV.

Same as Figure 2, we illustrate the quadratic electro-optic effect and electro-absorption process susceptibilities as functions of pump photon wavelength at 1.5-ps relaxation time in Figure 4. By comparing Figures 2 and 4, it is observed that the QEOEs and EA susceptibilities decrease and broaden with decreasing relaxation time.

**Figure 5:**
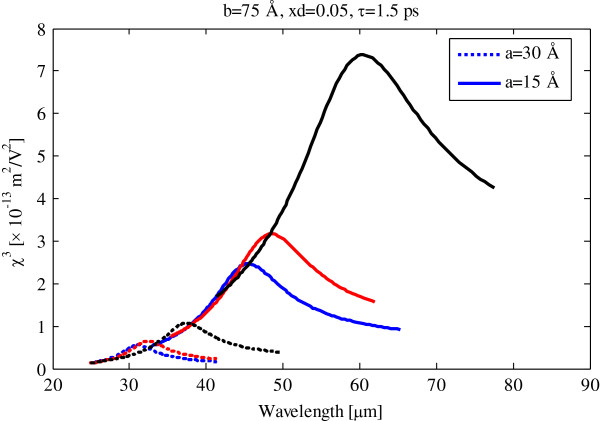
**Third-order susceptibility versus pump photon wavelength.** With different barrier potentials and defect sizes for 1.5-ps relaxation time (black *xb* = 0.1, red *xb* = 0.2, and blue *xb* = 0.3).

In Figure 5, we show the effect of confining quantum dot potential on third-order susceptibility. As can be seen with increasing barrier potential, the third-order susceptibility is decreased and blueshifted. Full-width at half maximum (FWHM) of third-order susceptibility in Figure 5 is approximately ten times broader than the FWHM in Figure 3.

**Figure 6:**
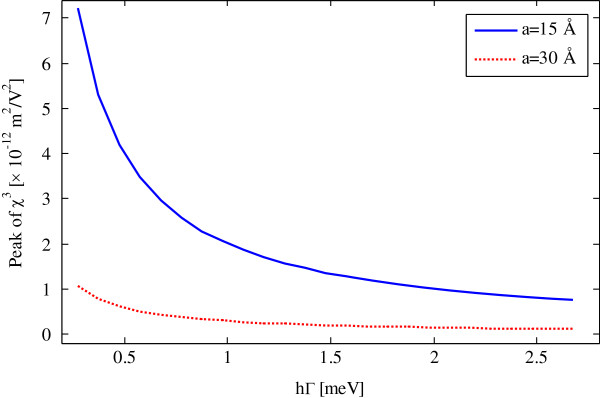
**Peak of third-order susceptibility as a function of relaxation constant.***b* = 75 Å, *xb* = 0.1, and *xd* = 0.05.

The effect of relaxation constant (*ħ*Γ) is demonstrated for two well sizes in Figure 6. It can be seen that the peak of the third-order susceptibility is decreased by the increase of the relaxation rate. It is clear from Equation 11 that the third-order susceptibility has an inverse relationship with relaxation constant. Also, the difference between the peak of susceptibilities in *a* = 15 Å and *a* = 30 Å is decreased with the increase of relaxation rate.

## Conclusions

In this paper, we have introduced spherical centered defect quantum dot (SCDQD) based on GaN composite nanoparticle to manage electro-optical properties. We have presented that the variation of system parameters can be tuned by the magnitude and wavelength of quadratic electro-optic effects and electro-absorption susceptibilities. For instance, the results show an increase of well width from 15 to 30 Å; the peaks of the both QEOEs and EA susceptibilities are decreased $\left(7.218\times 10^{-12}\frac{m^{2}}{V^{2}}\;\mathrm{to}1.062\times 10^{-12}\frac{m^{2}}{V^{2}}\right)$ and blueshifted (59.76 to 37.29 μm). With decreasing dot potential, the third-order susceptibility is increased $\left(2.444\times 10^{-12}\frac{m^{2}}{v^{2}}\;\mathrm{to}\;7.218\times 10^{-12}\frac{m^{2}}{v^{2}}\right)$ and red shifted (45.25 to 59.76 μm). The effect of relaxation constant (*ħ*Γ) which is verified by the peak of the third-order susceptibility is decreased by the increasing relaxation rate. These behaviors can be related to the quantum confinement effect and inverse impact of relaxation constant.

## Abbreviations

EA: electro-absorption; FWHM: full-width at half maximum; LEDs: light-emitting diodes; QEOEs: quadratic electro-optic effects; SCDQD: spherical centered defect quantum dot

## Competing interests

The authors declare that they have no competing interests.

## Authors' contributions

MK conceived of the study and participated in its design and coordination. AV assisted in the numerical calculations. AA and YH participated in the sequence alignment and drafted the manuscript. SWJ supervised the whole study. All authors read and approved the final manuscript.
